# Feasibility and Response to the San Diego County, California, Supplemental Nutrition Assistance Program (SNAP) Agency Sending Food and Nutrition Text Messages to All Participants: Quasi-Experimental Web-Based Survey Pilot Study

**DOI:** 10.2196/41021

**Published:** 2023-04-19

**Authors:** Wendi Gosliner, Celeste Felix, Ron Strochlic, Shana Wright, Allison Yates-Berg, Hannah R Thompson, Hao Tang, Blanca Melendrez

**Affiliations:** 1 Nutrition Policy Institute Division of Agriculture and Natural Resources University of California Oakland, CA United States; 2 Center for Community Health Altman Clinical and Translational Research Institute University of California San Diego San Diego, CA United States; 3 ideas42 New York, NY United States; 4 Health and Behavior Studies Teachers College Columbia University New York, NY United States

**Keywords:** SNAP, CalFresh, text, SMS text messaging, nutrition, fruits, vegetables, mHealth

## Abstract

**Background:**

The Supplemental Nutrition Assistance Program (SNAP) provides over 40 million Americans with money for food without typically providing participants with food or nutrition information. Educational SMS text messages can reach large numbers of people, and studies suggest SNAP participants appreciate nutrition education and have access to mobile phones.

**Objective:**

Using a pre-post intervention design, we assessed the feasibility of, and program satisfaction and outcomes resulting from, the San Diego County, California SNAP agency sending monthly food and nutrition education SMS text messages to all SNAP participants to increase fruit and vegetable purchasing and consumption.

**Methods:**

We developed and sent 5 behavioral science–informed SMS text messages with links to a project website in English and Spanish with information about selecting, storing, and preparing seasonal fruits and vegetables. The San Diego County SNAP agency sent monthly texts to ~170,000 SNAP households from October 2020 to February 2021. SNAP participants completed web-based surveys in response to a text invitation from the SNAP agency in September 2020 (baseline, n=12,036) and April 2021 (follow-up, n=4927). Descriptive frequencies were generated, and adjusted multiple linear mixed models were run on a matched data set of participants that completed both baseline and follow-up surveys (n=875) assessing pre- or postattitudes, behaviors, knowledge, and self-efficacy. We used adjusted logistic regression models to assess differences between the matched (n=875) and nonmatched (n=4052) participants related to experiences with the intervention (questions asked only at follow-up).

**Results:**

After the intervention, matched participants reported significant increase in knowing where to get information about selecting, storing, and preparing fruits and vegetables (3.76 vs 4.02 on a 5-point Likert scale with 5=strongly agree, *P*<.001); feeling good about participating in SNAP (4.35 vs 4.43, *P*=.03); and thinking the CalFresh program helps them eat healthy (4.38 vs 4.48, *P*=.006). No significant pre- or postdifferences were found in fruit or vegetable consumption, though most participants at follow-up (n=1556, 64%) reported their consumption had increased. Among the sample that completed the follow-up survey only (n=4052, not including 875 participants who completed follow-up and baseline), 1583 (65%) and 1556 (64%) reported purchasing and eating more California-grown fruits and vegetables, respectively. Nearly all respondents appreciated the intervention (n=2203, 90%) and wanted it to continue (n=2037, 83%).

**Conclusions:**

SNAP can feasibly provide food and nutrition messages via text to participants. A monthly text campaign was well received by responding participants and improved some measures of their self-reported knowledge, self-efficacy, produce consumption, and perceptions of SNAP participation. Participants expressed interest in continuing to receive texts. While educational messages will not solve the complex food and nutrition challenges confronting SNAP participants, further work should employ rigorous methods to expand and test this intervention in other SNAP programs before considering to implement it at scale.

## Introduction

A diet rich in fruits and vegetables can reduce the risk of cardiovascular disease, type 2 diabetes, some cancers, and obesity [1]. Despite the benefits of fruit and vegetable consumption, only 1 in 10 adults in the United States eat the daily recommended amounts of fruits and vegetables [2]. Consumption is even lower among individuals with low incomes, who face additional barriers including cost, limited availability, access, and often lack of time to cook and prepare fresh produce [3]. Studies find that participants in the Supplemental Nutrition Assistance Program (SNAP), like all Americans, consume fewer fruits and vegetables than the amount recommended by the Dietary Guidelines for Americans [3,4].

SNAP is the largest federal nutrition assistance program, with over 41 million Americans participating in an average month in 2021, a 4% increase from 2020 [5]. Approximately 4.5 million Californians participate in SNAP, known as CalFresh in California, to support their food purchases [6]. SNAP participants report a desire to eat a healthier diet, yet most consume less than the amount of fruits and vegetables recommended by the Dietary Guidelines for Americans [3,4]. While SNAP provides financial resources to support food purchases, the program does not typically provide food or nutrition information or any nutrition education. The Supplemental Nutrition Assistance Program-Education (SNAP-Ed) is intended to complement SNAP and provide people eligible for SNAP benefits with nutrition education as well as support to make physical and social environments more health-promoting [7]. However, SNAP-Ed programs typically are not specifically linked to SNAP agencies and typically reach only a small subset of SNAP participants each year, less than 10% in California, leaving most SNAP participants without access to consistent program-related information about food and nutrition [8].

Modern technologies offer efficient and relatively inexpensive mechanisms for delivering health messages to large population groups. Recent reports suggest that nearly all Americans (97%) own a cell phone, even among those earning less than $30,000 annually [9], making SMS text messaging a feasible way to deliver information to large numbers of people. SMS text messaging has been successfully utilized to promote health on diverse topics including nutrition, physical activity, tobacco cessation, and chronic disease management [10-14].

Studies have shown that SMS text message–based interventions can be effective in promoting behavior changes, such as healthy food purchasing habits, increased availability of fruits and vegetables in the home, greater self-efficacy for vegetable consumption, improved parent or caregiver health behavior modeling, reduced sugar-sweetened beverage consumption, and increased physical activity [15-19]. Studies of website-based interventions promoting improved diet and nutrition have shown promising results as well [20-22]. Components of SMS text messaging that have been found to increase effectiveness include personalization, tailoring and targeting messages based on demographic and psychosocial characteristics, and decreasing message frequency over time [13,22]. Some studies suggest that combining SMS text messages with educational websites to promote improved dietary behaviors increased effectiveness [23], although others have not found significant differences [10].

Using SMS text messaging systems could be a cost-effective way to reach participants in large-scale public health–related programs with information about health behaviors. However, there do not appear to be any studies published that report on research utilizing SMS text messaging systems to send food and nutrition information to all SNAP participants within the agency administering the program (in California, SNAP is administered at the county social services agency level; most states administer the program statewide) [24,25]. A few studies examining the effectiveness of SMS text message–based interventions promoting improved nutrition among SNAP and SNAP-eligible participants have reported positive findings [18,26]; however, interventions were implemented by outside organizations rather than integrating the information into the SNAP program. Findings from an intervention called Txt4HappyKids that targeted parents, including but not limited to SNAP participants, did not find statistically significant changes in behavior, self-efficacy, or attitudes related to fruit and vegetable intake from pre- to postintervention, although parents felt positive about the program and reported making changes to promote a healthier diet [26]. Another intervention that delivered 2 to 3 daily SMS text messages and a weekly newsletter to promote weight loss among 104 SNAP participants and SNAP-eligible women over 12 weeks found significant improvements in dietary and physical activity behaviors, food environment, goal setting, and reduced body weight [18].

The County of San Diego Health and Human Services Agency (HHSA), the SNAP agency in San Diego County, California, utilizes an opt-out SMS text message system to provide administrative reminders and alerts to their CalFresh participants. Our intervention was incorporated into this already existing system, from which CalFresh participants may opt-out at any time. This paper reports on a pilot intervention consisting of monthly SMS text messages promoting increased fruit and vegetable consumption through an existing default opt-out SMS text messaging system, with links to a website offering additional information and resources. While effective text interventions often include more frequent messaging, one text monthly was determined to be feasible for the host agency and respectful to participants who were not actively opting-in to participate; this pilot study was designed to assess whether this limited intervention was feasible and effective.

## Methods

### Study Design

The intervention was evaluated using a one-group pre-post study design. HHSA sent a single SMS text message inviting all CalFresh participants to complete a brief web-based survey at baseline and sent another single SMS text message to all CalFresh participants inviting them to complete a survey at follow-up. The survey assessed behaviors, attitudes, and self-efficacy related to fruit and vegetable intake and attitudes toward SNAP, seasonal fruits and vegetables, California-grown fruits and vegetables, and the SMS text message campaign. The survey was self-administered in English and Spanish. Survey participants were offered a free hard-copy cookbook in English or Spanish as a “thank you” for participation in the baseline survey. Follow-up survey participants were offered digital informational packets including resources, tips, and recipes on a range of topics related to healthy eating and cooking. One-time invitations to participate in baseline and follow-up surveys were sent to all SNAP participants in San Diego County who had not opted out of the SMS text message system, which included 176,162 participants in September 2020 (baseline) and 171,045 participants in April 2021 (follow-up).

### Ethics Approval

The institutional review boards of the University of California-Davis (protocol ID 1537832-1) and the University of California-San Diego (protocol ID 200239) approved all aspects of this study. The web-based survey included an informed consent section with a brief description of the research objectives and statements that participation is voluntary and confidential, personal information would be kept separate from other responses, nonparticipation would not affect participants’ relationship with the University of California or CalFresh/SNAP, and they would not directly benefit from the survey.

### Intervention

Research partners included a nonprofit (author initials blinded for peer review) that applies behavioral science insights to improve public programs. Together, a series of 5 SMS text messages promoting California-grown fruits and vegetables were developed. First, an academic literature review was conducted on the use of SMS text messaging to promote fruit and vegetable intake. A set of draft text messages were developed using best practice guidelines recommended for health-related behaviors [27,28]. Our SMS text messages were designed using evidence-based behavior change techniques, including reducing aversion to the cost of buying new fruits by explaining the benefits and consequences of fruit and vegetable intake (ie, providing pros and cons and persuasive argumentation); reducing ambiguity of in-season fruits and vegetables and cost misperceptions by highlighting cost-savings associated with fruit and vegetable intake and seasonal and local purchases (ie, shaping knowledge); providing actionable messages with links to next steps and a credible website to make access easy (ie, action planning); and promoting a feeling of benefiting others (in this case, supporting local growers; ie, providing social support and identification of self as a role model) [29,30]. English text messages were translated to Spanish by a bilingual, native Spanish-speaking staff member of the behavioral science partner organization and reviewed by multiple bilingual research team members. Next, we conducted 3 in-person focus groups with a total of 15 CalFresh participants in San Diego in March 2020, including 2 focus groups with 9 and 4 Spanish-speaking participants, respectively, and 1 with 3 English-speaking participants, to get insights about the draft messages and the intended intervention. The focus group participants provided information about their fruit and vegetable consumption and their selection, storage, and preparation of fruits and vegetables, interest in receiving food and nutrition information by text from CalFresh, and opinions about a sample set of draft text messages.

The Altman Clinical and Translational Research Institute Center for Community Health (CCH) at the University of California San Diego (UCSD) is the backbone organization for the San Diego County Childhood Obesity Initiative (COI). In the last step of SMS text message review, under COI’s Community Domain, a Community Advisory Board comprised of resident leaders provided feedback on a revised set of draft text messages and reviewed all translations. A final set of SMS text messages was developed, including an introductory message from HHSA and UCSD informing participants about the intervention, and five subsequent messages highlighting topics related to fruits and vegetables, including (1) the importance and safety of fruit and vegetable intake during the COVID-19 pandemic and California wildfire season, (2) the health benefits of different fruits and vegetables, (3) cost-savings associated with seasonal and local produce, (4) tips to try less familiar fruits and vegetables such as kiwis and persimmons, and (5) the benefits of shopping locally for farmers (Table 1). All SMS text messages were available in English and Spanish. The HHSA participant database includes a field for preferred language. All participants received SMS text messages in English, unless they indicated a preference for Spanish. HHSA distributed the SMS text messages monthly from September 2020 to February 2021.

**Table 1:** Monthly food and nutrition information SMS text messages sent by the County of San Diego Health and Human Services Agency to all Supplemental Nutrition Assistance Program (SNAP) participants in the feasibility study.

Month	Text message	Behavioral barriers addressed	Promising behavioral practices applied to messaging
September	Hello from CalFresh^a^ and UCSD^b^! We’ll be sending you monthly tips on fruits and veggies. Take this short, optional survey at Eat-CA.org/survey and get a recipe book!	Lack of trust in messenger’s authority and knowledge	Establishing sender as a credible source Setting expectations
October	Hello from CalFresh and UC San Diego! Fruits and vegetables are important (and safe!) to eat during COVID-19 and wildfire season. See here for recipes and tips: eat-ca.org/en	COVID-19 (misperceptions about risk)	Shaping knowledge: highlighting benefits instead of repeating misinformation
November	Hello from CalFresh and UC San Diego! Buying California-grown fruits and veggies supports local farmers and keeps your family healthy. Did you know that persimmons promote heart health, support healthy vision & reduce inflammation? Find out more: eat-ca.org/persimmons	Aversion to spending more on a new fruit and risking it going to waste COVID-19 (impact on local farmers)	Reducing aversion: highlighting benefits of trying a new fruit Providing social support or identifying self as a role model: feeling good about supporting local community
December	Hello from CalFresh and UC San Diego! Choosing in-season fruits and veggies can save you money. Buy beets in winter and save. Try them steamed, roasted or raw. You can eat the greens too! Learn more here: eat-ca.org/beets	Cost misperceptions and ambiguity about in-season fruits and vegetables	Providing clear and actionable guidance on in-season fruits and vegetables and cost savings Shaping knowledge: highlighting benefits of buying in-season fruits and vegetables
January	Hello from CalFresh and UC San Diego! Did you know kiwis have a sweet but unique flavor? Slice them and eat like an apple. YUM! You can even eat the skin! For recipes visit: eat-ca.org/kiwi	Aversion to spending money on a new fruit and risking it going to waste	Reducing aversion: highlighting benefits of trying a new fruit Providing clear and actionable guidance on how to prepare fruits and vegetables
February	Hello from CalFresh and UC San Diego! Did you know leafy greens help the body grow strong bones and teeth because they contain calcium? Include more California-grown fruits and vegetables in your meals and snacks. See here for ideas: eat-ca.org/leafy-greens	Ambiguity around health benefits of fruits and vegetables	Shaping knowledge: highlighting benefits of vegetables Providing clear and actionable guidance on how to prepare fruits and vegetables

^a^“CalFresh” is the name of the SNAP program in California.

^b^UCSD: University of California San Diego.

Each SMS text message included a link to a bilingual English-Spanish website developed by the project team and hosted by the UCSD Altman Clinical and Translational Research Institute. The website provided information about selecting, storing, and preparing seasonal California-grown fruits and vegetables. Draft content was reviewed by the behavioral science experts to ensure it was consistent with the behavioral science principles highlighted above. The website [31] was organized by season, with the current season being at the top at all times. Each season included individual webpages for 6 different seasonal fruits and vegetables, for a total of 24 fruits and vegetables with their own web pages. Each web page for a fruit or vegetable included user-friendly tips for selecting, storing, and preparing that produce item, information on the health benefits of eating that item, tips to extend the life of the item and reduce food waste, and user-friendly recipes incorporating the item, in both text and video formats. The web pages also included links to other websites offering additional resources and recipes, including EatFresh.org [32] (a website hosted by the California Department of Social Services) and the California Department of Public Health, CalFresh Healthy Living program [33]. Feedback on the draft website content was provided by the COI Community Advisory Board under the UCSD Altman Clinical and Translational Research Institute prior to the site’s launch.

### Instruments

#### Overview

The baseline and follow-up surveys were administered on the internet using Qualtrics. The questionnaires were designed to take participants about 10 minutes to complete. The baseline and follow-up surveys included 11 questions related to attitudes, knowledge, self-efficacy, behaviors, and perceptions of SNAP. The follow-up survey included an additional 9 questions assessing respondent perceptions of the intervention. Two questions assessing self-reported intake were adapted from the University of California Food Behavior Checklist [34], and 2 questions about the perceived importance of seasonal and California-grown fruits and vegetables were adapted from the University of California [35] and the University of Nebraska [36]. All other questions were developed by the research team.

#### Attitudes

The baseline and follow-up surveys measured attitudes with three statements: (1) It is important to eat a wide variety of fruits and vegetables (2) When choosing fruits and vegetables to buy, it is important to me to buy California-grown fruits and vegetables, and (3) When choosing fruits and vegetables to buy, it is important to me to buy fruits and vegetables that are in season (5-point scale, strongly disagree to strongly agree).

#### Knowledge and Self-efficacy

The baseline and follow-up surveys measured self-efficacy with four statements: (1) I know where to go to get information about selecting, storing, and preparing fruits and vegetables; (2) I am comfortable selecting, storing, and preparing a wide variety of fresh fruits; (3) I am comfortable selecting, storing, and preparing a wide variety of fresh vegetables; and (4) I want to learn more about how to prepare and eat a wide variety of fruits and vegetables (5-point scale, strongly disagree to strongly agree)*.*

#### Behavior

The baseline and follow-up surveys assessed self-reported fruit and vegetable intake with two questions: (1) How often do you eat 2 or more different kinds of fruits a day? and (2) How often do you eat 3 or more different kinds of vegetables a day? (5-point scale, never-always). At follow-up, participants were asked how strongly they agreed with statements assessing intent, intake, and purchasing: (1) I thought about eating fruits and vegetables more after getting the SMS text messages from CalFresh and UCSD; (2) I ate more fruits and vegetables after getting the SMS text messages from CalFresh and UCSD; and (3) I bought more California-grown fruits and vegetables after getting the SMS text messages from CalFresh and UCSD (5-point scale, strongly disagree to strongly agree).

#### Engagement and Satisfaction With the Intervention

The follow-up survey included 6 additional questions assessing user engagement and satisfaction with the intervention. The following two questions assessed engagement: (1) Do you remember getting any SMS text messages about fruit and vegetables from CalFresh and UCSD? and (2) Did you click on the website link included in the SMS text messages from CalFresh and UCSD? The questions assessing satisfaction with the SMS text messages were (1) I liked getting the SMS text messages about fruits and vegetables from CalFresh and UCSD and (2) I learned something from the texts about fruits and vegetables from CalFresh and UCSD. The two questions that assessed perceptions of CalFresh (SNAP) and interest in receiving continued messages were (1) I appreciated CalFresh for sending me information about nutrition and (2) I would like CalFresh and UCSD to keep sending me SMS text messages about fruits and vegetables (Responses to all questions were on a 5-point scale, strongly disagree to strongly agree).

### Analysis

Three analytic data sets were created: (1) a matched sample data set, consisting of respondents who completed both baseline and follow-up surveys; (2) a baseline sample data set, consisting of respondents who completed the baseline survey only; and (3) a follow-up sample data set, consisting of respondents who completed the follow-up survey only. Unpaired *t* tests were used to examine demographic differences between participants in the baseline only, follow-up only, and matched sample data sets. Multiple linear mixed models were used with time as the primary predictor and a random effect for participant, adjusted for gender identity, race and ethnicity, and survey language to assess pre-post changes on 11 survey questions among the matched sample data set. Additionally, to investigate whether there were any differences in outcomes for individuals who reported visiting the website compared to those who did not, we ran multiple linear mixed models with a visiting the website (yes or no) by time (baseline or follow-up) interaction term, adjusted for gender identity, race and ethnicity, and survey language, and a random effect for participant ID. To explore differences in responses between the matched and nonmatched individuals for the 9 follow-up questions assessing participants’ experiences with the intervention, we used logistic regression models adjusted for gender identity as well as a single variable representing race and ethnicity.

## Results

### Participant Characteristics

**Figure 1:**
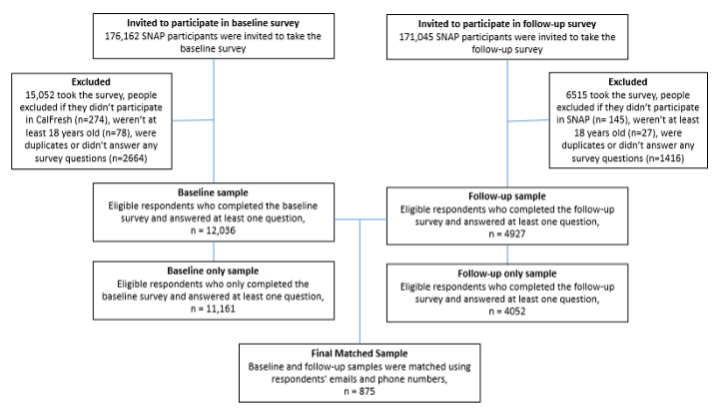
Final sample flowchart for the SNAP behavioral science–informed food and nutrition SMS text message intervention pilot. SNAP: Supplemental Nutrition Assistance Program.

We received 15,062 responses to the baseline survey (response rate 9%). Respondents 18 years or younger of age, not participating in CalFresh at the time, or those not answering any survey questions were excluded from analysis, leaving a baseline sample size of 12,036. At follow-up, we received 6515 responses (response rate 4%) for a follow-up sample data set size of 4927 after exclusion criteria were applied (Figure 1). We created a matched sample data set by matching baseline or follow-up emails or telephone numbers (n=875). In total, 11,161 participants completed the baseline survey only, and 4052 completed the follow-up survey only. Compared to both baseline only and follow-up only respondents, matched sample respondents were more likely to be female (*P*<.001) and White (*P*<.001 and *P*=.002, respectively; Table 2).

**Table 2:** Demographic characteristics, baseline, follow-up, and matched sample respondents^a^ in the Supplemental Nutrition Assistance Program behavioral science–informed food and nutrition SMS text message intervention pilot.

Characteristic			Baseline-only, n/N (%)		Follow-up only, n/N (%)		Matched, n/N (%)		*P* value for baseline vs matched		*P* value for follow-up vs matched
**Gender identity**											
	Female	6283/10,800 (58.2)		1891/3532 (53.5)		544/875 (62.2)		<.001		<.001	
	Male	4462/10,800 (41.3)		1583/3532 (44.8)		316/875 (36.1)		<.001		<.001	
	Prefer not to state, nonbinary, transgender, or other	55/10,800 (0.5)		58/3532 (1.6)		15/875 (1.7)		<.001		.88	
**Race and ethnicity**											
	American Indian or Alaska Native	103/10,752 (1.0)		28/3513 (0.8)		4/875 (0.5)		.04		.21	
	Asian or Asian American	660/10,752 (6.1)		221/3513 (6.3)		33/875 (3.8)		<.001		<.001	
	Black or African American	823/10,752 (7.7)		258/3513 (7.3)		50/875 (5.7)		.02		.07	
	Native Hawaiian or Pacific Islander	80/10,752 (0.7)		29/3513 (0.8)		4/875 (0.5)		.24		.18	
	White	3355/10,752 (31.2)		1211/3513 (34.5)		352/875 (40.2)		<.001		.002	
	Latino or Latina	4259/10,752 (39.6)		1244/3513 (35.4)		313/875 (35.8)		.02		.84	
	Checked more than one	818/10,752 (7.6)		288/3513 (8.2)		80/875 (9.1)		.13		.38	
	Other, do not know, or not sure	654/10,752 (6.1)		234/3513 (6.7)		39/875 (4.5)		.03		.01	
**Survey language**											
	English	8279/11,161 (74.2)		3039/4052 (75.0)		652/875 (74.5)		.83		.76	
	Spanish	2882/11,161 (25.8)		1013/4052 (25.0)		223/875 (25.5)		.83		.76	

^a^*t* test analyses conducted to identify differences between the baseline only and matched samples and between follow-up only and matched sample.

### Baseline and Follow-up Differences Among Matched Participants

There were statistically significant differences between baseline and follow-up with respect to 3 items: “I know where to go to get information about selecting, storing, and preparing fruits and vegetables” (*P*<.001), “the CalFresh program helps me eat healthy” (*P*=.006), and “I feel good about participating in CalFresh” (*P*=.04). Two other items had *P* values that approached, but did not reach, statistical significance (*P*=.05 to .06), while the other 5 items had no differences (Table 3). We did not find any statistically significant differences in outcomes between individuals who reported visiting the website and those who did not (data not shown).

**Table 3:** Changes in pre- or postattitudes, self-reported fruit and vegetable intake, knowledge, self-efficacy, and perceptions of Supplemental Nutrition Assistance Program (SNAP) among matched pairs in the SNAP behavioral science–informed food and nutrition SMS text message intervention pilot (N=875 matched pairs).

Measure		Adjusted baseline, mean (SE)	Adjusted follow-up, mean (SE)	Adjusted mean difference	*P* value^a^ (95% CI)
**Attitudes**					
	It is important to eat a wide variety of fruits and vegetables^b^	4.16 (0.12)	4.25 (0.12)	0.09	.08 (−0.01 to 0.18)
	When choosing fruits and vegetables to buy, it is important to me to buy fruits and vegetables that are in season^b^	4.11 (0.09)	4.13 (0.09)	0.02	.62 (−0.05 to 0.09)
	When choosing fruits and vegetables to buy, it is important to me to buy California-grown fruits and vegetables^b^	4.08 (0.10)	4.15 (0.10)	0.07	.05 (<−0.001 to 0.15)
**Knowledge and self-efficacy**					
	I know where to go to get information about selecting, storing, and preparing fruits and vegetables^b^	3.76 (0.12)	4.02 (0.12)	0.26	<.001 (0.17 to 0.34)
	I am comfortable selecting, storing, and preparing a wide variety of fresh fruits^b^	4.03 (0.11)	4.10 (0.11)	0.07	.50 (<−0.001 to 0.16)
	I am comfortable selecting, storing, and preparing a wide variety of fresh vegetables^b^	3.98 (0.11)	4.01 (0.11)	0.03	.43 (−0.05 to 0.11)
	I want to learn more about how to prepare and eat a wide variety of fruits and vegetables^b^	4.32 (0.09)	4.30 (0.09)	−0.02	.61 (−0.09 to 0.05)
**Consumption**					
	Eat 2 or more different kinds of fruit a day^c^	3.74 (0.11)	3.80 (0.11)	0.06	.06 (−0.003 to 0.11)
	Eat 3 or more different kinds of vegetables a day^c^	3.59 (0.11)	3.60 (0.11)	0.01	.88 (−0.06 to 0.07)
**Perceptions of CalFresh** ^d^					
	I feel good about participating in CalFresh^b,d^	4.39 (0.09)	4.47 (0.09)	0.08	.04 (0.002 to 0.14)
	The CalFresh program helps me eat healthy^b,d^	4.38 (0.10)	4.48 (0.10)	0.10	.006 (0.03 to 0.17)

^a^Values from multiple linear mixed models with time as the primary predictor and a random effect for participant, adjusted for gender identity, race and ethnicity, and survey language.

^b^Response options ranged from 1 (strongly disagree) to 5 (strongly agree).

^c^Response options ranged from 1 (never) to 5 (always).

^d^“CalFresh” is the name of the SNAP program in California.

### Intervention Perceptions Among Matched and Nonmatched Follow-up Participants

Using data from the follow-up data set, nearly two-thirds of respondents reported clicking on a link to the intervention website that was included in the SMS text messages with over half of those visiting the website reporting doing so two or more times (Table 4). Two-thirds of respondents reported buying and eating more fruits and vegetables after receiving the SMS text messages. Nearly all respondents reported liking the intervention and wanting it to continue. Responses to perceptions of the intervention were more strongly favorable among the matched population than among the entire population responding. For example, 86% (n=614) of matched participants agreed that they learned something from the texts compared to 77% (n=1880) of follow-up only participants.

**Table 4:** Intervention exposure, self-reported behavior changes, and intervention perceptions in the Supplemental Nutrition Assistance Program (SNAP) behavioral science–informed food and nutrition SMS text message intervention pilot; follow-up survey only respondents and matched sample (N=4927).

Measure			Follow-up survey only respondents (N=4052),^a^ n (%)		Matched sample (N=875),^b^ n (%)		*P* value^c^ (95% CI)
**Exposure to the intervention**							
	Remembered getting texts about fruits and vegetables from CalFresh^d^	2558 (65)		712 (82)		<.001 (0.76 to 1.88)	
	Clicked on the website link included in the SMS text messages from CalFresh	1533 (60)		503 (71)		.12 (−0.14 to 1.31)	
	Of those who visited the website, reported going to the website 2 or more times	813 (56)		324 (65)		.70 (−1.23 to 0.82)	
**Reported behavior change**							
	Ate more fruits and vegetables after getting the SMS text messages (agree or strongly agree)	1556 (64)		487 (68)		.16 (−0.21 to 1.24)	
	Bought more California-grown fruits and vegetables after getting the SMS text messages (agree or strongly agree)	1583 (65)		490 (69)		.08 (−0.09 to 1.37)	
**Perceptions of the intervention**							
	Liked getting the texts about fruits and vegetables (agree or strongly agree)	1922 (79)		626 (88)		.01 (0.24 to 1.85)	
	Learned something from the texts about fruits and vegetables (agree or strongly agree)	1880 (77)		614 (86)		<.001 (0.52 to 2.06)	
	Would like CalFresh to keep sending SMS text messages about fruits and vegetables (agree or strongly agree)	2037 (83)		658 (92)		.12 (−0.21 to 1.74)	
	Appreciated CalFresh for sending information about nutrition (agree or strongly agree)	2203 (90)		675 (95)		.44 (−0.72 to 1.67)	

^a^Due to survey design, not all questions were answered by 4052 participants.

^b^Due to survey design, not all questions were answered by 875 participants.

^c^*P* values from logistic regression adjusted for gender identity and race and ethnicity.

^d^“CalFresh” is the name of the SNAP program in California.

## Discussion

### Principal Findings

According to the US Department of Agriculture Food and Nutrition Service, “SNAP provides nutrition benefits to supplement the food budget of needy families so they can purchase healthy food and move towards self-sufficiency” [37]. While the program always provides financial resources for food purchases, SNAP programs do not systematically provide participants with access to information about healthy eating. SNAP-Ed programs operate somewhat differently in all states, but in no state are educational resources integrated into the SNAP program and made available to all participants.

Studies have shown that digital interventions incorporating SMS text messages or websites or a combination can be effective in promoting healthy behavior changes [15-22]. Some studies suggest that combining SMS text message interventions with educational websites to promote improved dietary behaviors increased effectiveness [23], although others have not found significant differences [10]. This study is the first in the nation that we know of to test an intervention providing digital food and nutrition information via SMS text messaging and a website to all county SNAP participants who had not opted out (unfortunately, opt-out data were not available from HHSA). While the scope of the pilot study was small, evaluating only 5 months of a monthly SMS text message, the results suggest this approach warrants longer and more rigorous evaluation. The fact that more than 15,000 people, representing nearly 10% of those responding to our initial survey suggests that many SNAP participants are reading the digital information they receive from the SNAP agency. Studies find that typically 54%-96% of SMS text message viewers engage with it by either reading, saving, sharing, or responding to the SMS text messages [38,39]; if that were true here, about 85,000 San Diego County SNAP participants would have read the SMS text messages they received as part of this pilot study. Our results suggest that at least a sizable subset of SNAP participants could benefit from this type of service being part of the program. Given that SNAP serves more than 40 million people annually [40], even if 5% of people benefitted from such a service, that would impact 2 million people. Further studies should assess the impacts and the costs and benefits of implementing food and nutrition SMS text messaging into SNAP programs.

Data we collected suggest at least some SNAP participants welcome receiving digital food and nutrition-related information from the SNAP agency. Nearly all respondents to our surveys appreciated the messages and wanted them to continue. Few survey respondents (4%) reported not appreciating the SMS text messages and 6% preferred not to continue receiving them (data not shown). While we may have missed people who disliked the intervention, studies suggest that it is typical that people who both very much like as well as those who very much dislike any intervention are likely to respond when asked to provide feedback [41]. Thus, while future studies should more rigorously assess perceptions of the intervention using a randomized controlled trial design, our results hold promise that the intervention may well appeal to a large number of participants.

Based on positive feedback from participants, the San Diego HHSA expressed interest in extending the SMS text message intervention beyond the pilot phase. Therefore, we developed an additional 16 messages with information on storing fruits and vegetables, reducing food waste and innovative ways to incorporate more fruits and vegetables. HHSA sent those messages between April 2021 and August 2022 and recently requested additional messages so that they can continue the intervention.

Our measure of change in fruit and vegetable consumption focused on whether participants ate 2 or more different kinds of fruits and 3 or more different kinds of vegetables per day. The responses to those questions at baseline reflected a high rate of people who said they did this almost always. Thus, there was not as much room for improvement in this measure as may have been needed to assess change. Using a more sensitive measure of fruit and vegetable consumption would be important in future studies. While our study did not find changes in our pre- compared to postfruit and -vegetable consumption measure, most participants at follow-up self-reported that they both bought and ate more California-grown fruits and vegetables as a result of receiving the SMS text messages (n=1583, 65% and n=1556, 64% of all follow-up only respondents, respectively and n=490, 69% and n=487, 68% for matched responses, respectively). It is possible that many participants ate more servings or ate larger portions of fruits and vegetables after the intervention. Our pre- compared to postconsumption measure may not have been sensitive enough to pick this up. Again, more rigorous study of this intervention is warranted.

Some studies suggest that participating in safety net programs such as SNAP can be stigmatizing and frustrating for participants [42]. These feelings can be exacerbated by efforts to restrict SNAP shoppers from using their benefits to purchase foods and beverages that are considered unhealthy [43,44]. SNAP participants have also described negative experiences with SNAP caseworkers and agencies and subsequent feelings of stress, humiliation and being devalued [45]. In addition to the emotional toll of these experiences, they could negatively impact SNAP participation rates. Our pilot study sent SMS text messages intended to make participants feel good about their participation in SNAP. Our results suggest that we achieved this objective among the subset of participants completing our surveys. In our matched sample, we found significantly more favorable responses to the item, “I feel good about participating in CalFresh” at follow-up compared to baseline. Additionally, 90% (n=2203) of survey respondents responding to a post-only question reported appreciating CalFresh for offering information about nutrition.

### Limitations

Our pilot study was funded to be an intervention with limited resources for the evaluation study. Our cross-sectional data collection approach and posthoc matching of respondents provide insights about the pilot study but more rigorous sampling methods and study designs are needed. Further, our low survey response rate, which may result from only a single SMS text message being sent to invite SNAP participants to take the surveys at both time periods, makes the study vulnerable to selection bias. We expect that more engaged people were more likely to have completed the surveys. However, the San Diego HHSA was not able to send additional messages to increase the survey response rate. Additionally, the surveys were available in English and Spanish only, thereby excluding participants not fluent in either language.

Using a randomized controlled study design for a future study would offer significantly better understanding of any benefits or drawbacks of this type of SNAP digital intervention. Additionally, if future studies identify increased fruit and vegetable intake associated with this intervention, with no concomitant increase in SNAP benefits, it would be important to understand other changes participants may have made. Future studies should test the intervention in additional languages, rather than just English and Spanish.

Our follow-up survey response rate (4%) was considerably lower than our baseline survey response rate (9%). That may be due to the fact that we offered a hard-copy cookbook as an incentive for participation in the baseline survey but offered a digital resources packet as an incentive for participation in the follow-up survey due to unexpectedly high survey completion at baseline and inadequate budget to provide cookbooks again at follow-up. We based the analysis on people who responded at both baseline and follow-up to limit the potential bias due to the significant differences we found between the baseline and matched samples with respect to a number of demographic characteristics.

Our findings indicate that the matched sample, which represents a more engaged subset of SMS text message recipients, reported better outcomes and more positive perceptions of the intervention than follow-up survey respondents in general. Our findings about the effects of the intervention using the pre- or postdesign may therefore not be representative of all SMS text message recipients in San Diego County.

We were not able to assess unintended consequences of this intervention due to a lack of available data about participants who opt-out of receiving SMS text messages from HHSA. It would be beneficial for future studies to explore whether any participants disliked the health-related SMS text messages so much that they decided to opt-out of receiving administrative texts or whether any other harm was caused. A qualitative study of participants who opt-out of texts during the intervention period would provide more comprehensive understanding of program impact and could reduce attrition in future efforts.

### Conclusions

Overall, this pilot study suggests that sending SNAP participants monthly food and nutrition educational SMS text messages can benefit participants. Our intervention design model, which included working closely with members of the community to develop and test the intervention methods and modalities and utilizing behavioral science insights to craft messages, led to a well-received intervention among participants responding to our surveys. This study found reported pre- compared to postimprovements in knowing where to go to get information about how to select, store, and prepare fruits and vegetables as well as comfort levels in selecting, storing, and preparing fruits and vegetables. Nearly two-thirds of survey respondents self-reported at follow-up that they had increased their purchase and consumption of fruits and vegetables as a result of receiving the SMS text messages. Additionally, respondents reported feeling better about participating in SNAP at follow-up compared to baseline. While educational messages will not solve the complex food and nutrition challenges confronting SNAP participants, they may be helpful to some people. Further studies should use more rigorous methods to test this intervention in SNAP programs and assess any negative unintended consequences before considering implementing this type of intervention at scale.

## Abbreviations

CCH: Center for Community Health
COI: Childhood Obesity Initiative
HHSA: Health and Human Services Agency
SNAP: Supplemental Nutrition Assistance Program
SNAP-Ed: Supplemental Nutrition Assistance Program-Education
UCSD: University of California San Diego

## Data Availability

The data sets generated and analyzed during this study are available from the corresponding author on reasonable request.
